# Mountain chickadees from different elevations sing different songs: acoustic adaptation, temporal drift or signal of local adaptation?

**DOI:** 10.1098/rsos.150019

**Published:** 2015-04-29

**Authors:** Carrie L. Branch, Vladimir V. Pravosudov

**Affiliations:** Department of Biology and Graduate Program in Ecology, Evolution, and Conservation Biology, University of Nevada, Reno Max Fleischmann Agriculture Building, 1664 North Virginia Street, Reno, NV 89557, USA

**Keywords:** song dialects, mountain chickadee, local adaptation, acoustic adaptation, elevation gradient, continuous habitat

## Abstract

Song in songbirds is widely thought to function in mate choice and male–male competition. Song is also phenotypically plastic and typically learned from local adults; therefore, it varies across geographical space and can serve as a cue for an individual's location of origin, with females commonly preferring males from their respective location. Geographical variation in song dialect may reflect acoustic adaptation to different environments and/or serve as a signal of local adaptation. In montane environments, environmental differences can occur over an elevation gradient, favouring local adaptations across small spatial scales. We tested whether food caching mountain chickadees, known to exhibit elevation-related differences in food caching intensity, spatial memory and the hippocampus, also sing different dialects despite continuous distribution and close proximity. Male songs were collected from high and low elevations at two different mountains (separated by 35 km) to test whether song differs between elevations and/or between adjacent populations at each mountain. Song structure varied significantly between high and low elevation adjacent populations from the same mountain and between populations from different mountains at the same elevations, despite a continuous distribution across each mountain slope. These results suggest that elevation-related differences in song structure in chickadees might serve as a signal for local adaptation.

## 2. Introduction

In many songbird species, male song serves as an important cue in female mate choice [1,2] and may be used to defend territories from invasion by conspecific males [3]. Song is typically learned from other local males [4], is phenotypically plastic [5] and can be shaped by female preference [6]. Variation in song not only aids in species recognition, but is particularly useful for female mate choice because it allows females to discriminate between males from local versus non-local populations. Females have been shown to exhibit a preference for local males, which may be indicated by local dialects [7,8]. This preference may benefit females because males occupying the same local habitat may possess genetic material beneficial for survival at that specific location (local adaptation, e.g. [9]).

While song dialects or geographical variation in song within a species allow for identification of locally suited individuals, there are several mechanisms by which such variation could arise. The acoustic adaptation hypothesis suggests that song dialects or geographical variation in song may arise as a result of changes in the environment affecting signal transmission [10]. In this case, shifts in song could either evolve and become fixed or simply be adjusted accordingly by individuals, with functional significance such that signal transmission is optimized in a new environment (e.g. differences in plant density in different forests). The temporal variation hypothesis suggests that song may simply drift or change over time within a population with no particular adaptive significance and, therefore, different populations with limited gene flow might evolve different song dialects simply by drifting apart [11]. Finally, the local adaptation hypothesis suggests that individuals locally adapted to their environment may experience limited movement, and over time female preference for local males may produce divergence in song, creating different song dialects within a species [12,13]. These changes may serve as an additional cue for females to mate assortatively with males locally adapted to their location of origin (*sensu* individuals colonizing new areas and mistakes in males' song imitation resulting in new dialects [14]). The local adaptation hypothesis predicts that vocal dialects should evolve via sexual selection in heterogeneous environments regardless of spatial scale [15,16] when such heterogeneity is associated with local adaptations limiting movement [12,17]. However, evidence linking specific local adaptations—beyond acoustic adaptation—with song dialects is lacking [9].

Montane environments present an especially striking example of rapid environmental change over an elevation gradient, which is usually associated with highly predictable climatic changes. As such, individuals living along montane elevation gradients are predicted to vary phenotypically [18–21]. Likewise, songbirds would be predicted to have different song dialects associated with such elevation-specific adaptations, despite small spatial scale or lack of geographical barriers [13,15,17].

Here, we examined song variation associated with differences in elevation in food caching mountain chickadees (*Poecile gambeli*) from different elevations. Chickadees are resident songbirds inhabiting the mountains of western North America that form permanent flocks of unrelated individuals outside of the breeding season [22]. Chickadees are scatter-hoarders, caching large quantities of food in numerous locations during the autumn, to be consumed over winter when food is scarce [22]. Chickadees from different elevations vary across a number of behavioural and neural traits thought to reflect local adaptations to elevation-specific environments [23–26]. For example, birds occupying habitat at higher elevations experience harsher winter conditions (lower temperature, more snow, extended periods of snow cover [27]) and be likely to have a higher reliance on previously cached food to survive winter [23]. As such, chickadees exhibit large elevation-related differences in memory needed to retrieve caches associated with large differences in the hippocampus, a brain region known to be involved in spatial memory [23–25]. These differences were discovered in juvenile birds prior to their first winter and therefore prior to the period of largest climate-related mortality. In addition, recent data comparing high versus low elevation chickadees suggests group level behavioural types, such that high elevation males are slower explorers, less aggressive and socially subordinate to low elevation males [26,28]. What is most striking is that these differences exist on very a small spatial scale in a highly mobile species—chickadees from high and low elevations are separated by just a few kilometres with only 600 m difference in elevation.

Taken together, these data suggest that birds hatched at high elevations would be likely to fare poorly at low elevations, owing to their socially subordinate status, whereas low elevation birds would be likely to fare poorly at high elevations with harsher winter conditions, owing to their inferior memory limiting successful cache retrieval. As a result, it may be expected that females would benefit from selecting males from their respective elevations. Indeed, given a pairwise choice of a high or low elevation male, high elevation females preferred high elevation males, whereas low elevation females showed no preference [29]. It is unclear how high elevation females are able to discriminate between high and low elevation males; however, given the pervasiveness of geographical variation in the songs of songbirds as well as the role of song in mate choice [1,7], variation in song may be one cue females use to discriminate between males from high and low elevations.

Here we assessed the song structure of male mountain chickadees using two exemplars of high and low elevation habitats on two different mountains in the Sierra Nevada with similar mixed-conifer forest structure. The structure of male song collected from multiple individuals at four locations was compared using basic acoustic parameters: duration, frequency and amplitude. Sampling song from males at high and low elevations from two different mountains allowed us to (i) assess the presence of any differences in song structure at different elevations and (ii) consider the potential mechanism generating variation or song dialects on a small spatial scale. If there are any differences in song structure among these four locations, the acoustic adaptation hypothesis predicts that song should differ between but not within elevations, because the composition of the mixed-conifer forests in Sierra Nevada are fairly standard and do not vary significantly between sites at similar elevations [30] and should therefore shape the song in similar ways in order to optimize signal transmission. The temporal variation hypothesis predicts that songs are selectively neutral and may drift and change over time. If there is no movement between birds from different elevations and/or mountains, then song structure could be different at all four locations. Finally, the local adaptation hypothesis predicts that limited movement between high and low elevation birds due to local adaptations, as well as limited movement between mountains due to short dispersal distances, would result in differences in song structure among all four locations, resulting in local songs or dialects used by females to choose local males [31].

## 3. Material and methods

### 3.1 Subjects and song collection

Male mountain chickadee song was recorded from high and low elevation sites at two spatially distinct mountain slopes separated by approximately 35 km, Sagehen, CA (Mountain 1) and Mt. Rose, NV (Mountain 2), between 06.00 and 13.00 h PST from 13 April to 9 June 2013. The high elevation sites were approximately 2400 m and the low elevation sites were approximately 1800 m at both locations (following [23]). At Sagehen (Mountain 1), we recorded at exactly the same elevations and locations used previously [23–26,29]. Males in these areas are not individually colour banded; therefore to avoid duplicating subjects, we recorded males with a minimum of 500 m separation between sites [32]. A minimum of 20 songs from at least 12 males per site were recorded using a Marantz PMD661 Compact Flash Card digital recorder and Sennheiser ME-66 unidirectional microphone with a sampling rate of 44 000 Hz and 16 bit resolution. Males were located auditorily and approached with microphone and recorder in hand. Each male was recorded on 1 day.

### 3.2 Acoustic analysis

**Figure 1.**
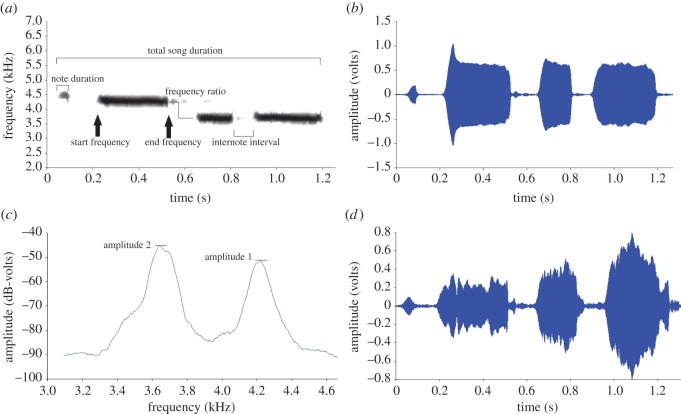
Schematic of sound spectrogram and power spectrum showing acoustic measurements used for analysis. Other measurements not physically shown in diagram were calculated using the measurements depicted. (*a*) Sound spectrogram (transform length of 512 points, time resolution of 11.6 ms, and frequency resolution of 86.1 Hz); measurements include temporal: total SD, ND, II and spectral: SF, end frequency (used with SF to calculate NG), change in frequency from end of Note 2 to start of Note 3 used to calculate FR, also the NN can be counted from the spectrogram, there are four notes in this example. (*b*) Waveform of 1a. (*c*) Power spectrum (FFT window=65 536 points; 88 Hz smoothing) showing amplitude 1 and 2 used to calculate RA. (*d*) Waveform of 1c.

Song recordings were first viewed in 10 s interval spectrograms from large .wav files using SIGNAL 5 digital signal analysis system (Engineering Design, Berkley, CA, USA); each song was then edited and saved in an individual .wav file. For each male (Mountain 1 low, *n*=15; Mountain 1 high, *n*=17; Mountain 2 low *n*=12; Mountain 2 high *n*=12), we analysed a minimum of six quality songs limited by amount of noise (range: 6–23 songs per male). To obtain a comprehensive description of the song, we extracted 19 acoustic measures based on previous work on mountain and black-capped chickadees (table 1 and figure 1) [33–35]. Mountain chickadee song typically consists of either three or four notes, with variation in the presence/absence of an introductory note [33]. We extracted measures from the entire song as well as the individual notes within each song; therefore, the number of measures per song was partially dependent on the presence/absence of the introductory note.

**Table 1.** Means and standard errors for each song characteristic measured from high and low elevation males at both Mountain 1 and Mountain 2 locations. Bold values represent significant difference between elevations within a location using Tukey post hoc comparisons at *p*<0.05. Italics indicate very closely approaching significant values, 0.056. Acoustic measures in bold and italics represent the 11 variables used in DFA.

	Mountain 1			Mountain 2		
acoustic measure	low	high	*p*-value	low	high	*p*-value
**NN**	3.85±0.08	3.85±0.08	0.999	**3.81**±**0.09**	**3.36**±**0.11**	**0.011**
N1 SF	4356.82±20.22	4388.43±23.50	0.828	4338.56±20.79	4357.82±56.17	0.976
N2 SF	4291.04±17.78	4306.97±23.62	0.999	4297.15±25.97	4288.22±32.64	1
**N3 SF**	**3503.35**±**18.78**	**3659.19**±**20.82**	¡**0.001**	3608.64±21.10	3611.20±21.79	1
N4 SF	**3590.68**±**16.41**	**3730.89**±**19.26**	¡**0.001**	3682.67±21.18	3660.37±20.03	0.999
**FR**	**705.23**±**20.64**	**591.33**±**18.10**	**0.005**	641.78±21.02	609.48±38.73	0.823
N1 G	0.99±0.003	0.99±0.002	0.993	**0.98**±**0.003**	**0.99**±**0.009**	**0.039**
*N*2 *G*	1.02±0.003	1.01±0.002	0.896	1.01±0.003	1.02±0.005	0.976
*N*3 *G*	0.99±0.003	0.1±0.003	0.275	0.99±0.003	0.99±0.003	0.999
*N*4 *G*	0.1±0.003	1.01±0.002	0.414	0.1±0.002	0.99±0.002	0.992
SD	1201.42±16.99	1129.55±19.11	0.079	1140.70±31.84	1094.03±21.39	0.514
N1 D	44.72±2.07	40.62±1.43	0.421	39.29±2.20	36.96±3.28	0.905
**N2 D**	**316.98**±**4.22**	**279.88**±**5.91**	¡**0.001**	303.89±7.74	290.68±5.33	0.839
**N3 D**	**188.73**±**2.78**	**160.10**±**2.21**	¡**0.001**	193.59±4.29	182.36±1.93	0.942
*N*4 *D*	294.23±5.73	279.82±4.17	0.539	278.61±6.58	301.18±5.89	0.106
N1–N2 II	***127*.*78***±***2*.*78***	***140*.*67***±***3*.*19***	***0*.*056***	127.26±3.06	119.41±8.38	0.614
N2–N3 II	138.12±3.06	140.46±3.88	0.999	138.68±4.37	145.77±6.00	0.931
**N3–N4 II**	114.84±3.13	109.35±2.67	0.962	***99*.*78***±***3*.*15***	***117*.*98***±***4*.*98***	***0*.*056***
**RA**	0.93±0.015	0.92±0.016	0.984	**0.96**±**0.016**	**0.86**±**0.015**	¡**0.001**

Temporal and spectral measures were taken from sound spectrograms: eight temporal measures including (i) total song duration (SD), (ii) note duration (ND; 3 or 4 depending on the song) and (iii) internote interval (II; 2 or 3 depending on song); and nine spectral measures including (i) start frequency for each note (SF; 3 or 4 depending on song), (ii) frequency ratio (FR; mountain chickadee songs have a frequency shift between Notes 2 and 3, where the frequency of the note drops (calculated by dividing the end frequency of Note 2 by the SF of Note 3)) and (iii) the glissando of each note (NG; 3 or 4 depending on the song, calculated by dividing the SF of the note by the end frequency of the note). We also measured the number of notes (NN) in each song. The one amplitude measure, (i) relative amplitude (RA), was taken from a power spectrum and calculated by dividing the maximum amplitude of Note 3 by the maximum amplitude of Note 2. Temporal and spectral measures were made using a spectrogram window size of 512 points, a time resolution of 11.6 ms and a frequency resolution of 86.1 Hz.

### 3.3 Statistical analysis

For each measure described, we calculated means and standard deviations across all songs produced by the same male. This way each bird is only represented once in all statistical analyses and pseudoreplication is avoided. We analysed SF, NG, ND and II of Notes 2, 3 and 4 using four repeated measures general linear models (GLMs; Statistica v. 12), where the acoustic measure from male song was the repeated measure (e.g. SF of Notes 2, 3 and 4) and elevation (high, low), location (Mountain 1, Mountain 2), and an interaction between elevation and location were the between-subject variables. Univariate GLMs were used to assess differences between male song at each of the four locations on NN, SD, FR, RA, as well as SF, NG, ND and II of birds with at least one song containing Note 1 (the introductory note). If a bird did not have the introductory note (Note 1), the analyses for that bird started with Note 2. Tukey posthoc tests were used for all pairwise comparisons, with significance established at *p*<0.05. In addition, we calculated location-specific individual variation using the coefficient of variation (CV) for each acoustic measure for every bird and used GLMs to assess how consistently male songs were produced (used to assess potential differences in male quality [36–38].

To further address differences in songs from these four locations, we ran two ‘all-variables-together’ discriminant function analyses (DFAs) (Statistica v. 12) to assess song classification by location. In an ‘all-variables-together’ DFA, all predictor variables are used at the same time to derive the discriminant functions model [39,40]. We ran two models using the same 11 predictor variables, one based on mean values and one based on CV values for each predictor. The number of variables in a DFA are limited by the least number of objects in a group minus one [39]; our lowest sample size was 12, therefore we used 11 predictor variables. Seven variables (NN, Note 3 SF, FR, Note 2 D, Note 3 D, Note 3–4 II, RA, see bolded variables in table 1) were chosen based on significant differences from GLMs and four (Note 4 D, Note 2 G, Note 3 G, Note 4 G, see italicized variables in table 1) were added to increase likelihood of discrimination. Location-specific individual variation was assessed by using the CV of each acoustic measure for each song within a birds' repertoire, therefore the CVs represent how consistently an individual produces its song [41].

We used the ‘hold-out-sample’ method to cross-validate the location classifications, which simply involves running a DFA without a set of observations from each group and then using those results to classify the observations that were held out [39,40]. For our analysis, we held out three randomly chosen males from each location (12 total). The same cases were held out for both DFAs.

## 4. Results

### 4.1 General linear models

#### 4.1.1 Temporal

**Figure 2.**
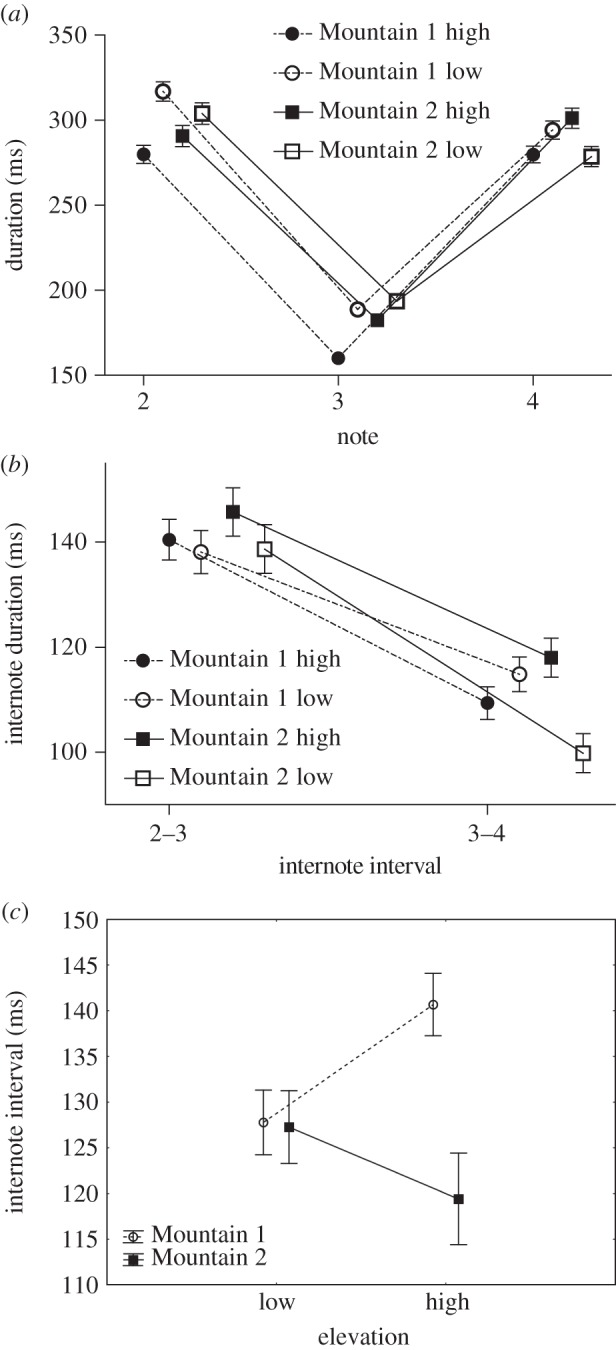
Means and s.e.m. for the three temporal measures that showed significant differences using Tukey post hoc comparisons. (*a*) ND for Notes 2, 3 and 4 from high and low elevation males sampled at Mountains 1 and 2, (*b*) II between Notes 2–3 and Notes 3–4 from high and low elevation males sampled at Mountains 1 and 2, (*c*) II between Notes 1–2 from high and low elevation males sampled at Mountains 1 and 2.

There was no significant effect of overall location (*F*_1,52_=1.93, *p*=0.171) for ND of Notes 2, 3 and 4, however there was a significant effect of elevation (*F*_1,52_=13.85, *p*<0.001), and significant interaction between location and elevation (*F*_1,52_=12.62, *p*<0.001; figure 2*a*). There was a significant difference in ND for Notes 2 and 3 between high and low elevation male song from Mountain 1, but no differences within Mountain 2 (table 1). Additionally, there were significant differences in Note 2 duration between Mountain 1 low and Mountain 2 high (*p*=0.012) and between Mountain 1 high and Mountain 2 low (*p*=0.026); and in Note 3 duration between Mountain 1 high and Mountain 2 low (*p*<0.001), and Mountain 1 high compared with Mountain 2 high approached significance (*p*=0.059).

There was no effect of overall location (*F*_1,52_=0.002, *p*=0.967) or elevation (*F*_1,52_=2.66, *p*=0.109) on the II between Notes 2 and 3 or Notes 3 and 4, however there was a significant interaction between location and elevation (*F*_1,52_=4.40, *p*=0.041; figure 2*b*). There were significant differences between the II of Notes 3 and 4 for high and low elevation males at Mountain 2, but no significant differences at Mountain 1 (table 1). No other pairwise comparisons were significant between mountains.

There was a significant effect of overall location (*F*_1,52_=4.66, *p*=0.036) and elevation (*F*_1,52_=7.06, *p*=0.010) on SD, however the interaction between location and elevation was not significant (*F*_1,52_=0.32, *p*=0.575). There were no significant differences between high and low elevation males' SD at Mountain 1 or Mountain 2 (table 1). However, there was a significant difference between the SD at Mountain 1 low and Mountain 2 high (*p*=0.008).

Further analyses of individuals with at least one song with the introductory note (Mountain 1 low *n*=14; Mountain 1 high *n*=15; Mountain 2 low *n*=11; Mountain 2 high *n*=7) showed an effect of overall location (*F*_1,43_=4.35, *p*=0.043) on Note 1 ND, however there was no significant effect of elevation (*F*_1,43_=2.184, *p*=0.147), and the interaction between location and elevation was not significant (*F*_1,43_=0.16, *p*=0.688). There were no significant differences between high and low elevation male Note 1 ND at Mountain 1 or Mountain 2 (table 1) or between mountain comparisons.

There was a significant effect of overall location (*F*_1,43_=7.29, *p*=0.01), but no effect of elevation (*F*_1,43_=0.39, *p*=0.535) on II between Notes 1 and 2, however there was a significant interaction between location and elevation (*F*_1,43_=6.60, *p*=0.014; figure 2*c*). The difference in Note 1 II between high and low elevation birds at Mountain 1 approached significance, but there was no difference at Mountain 2 (table 1). There was a significant difference in Note 1 II between Mountain 1 high and Mountain 2 high (*p*=0.006).

#### 4.1.2 Spectral

**Figure 3.**
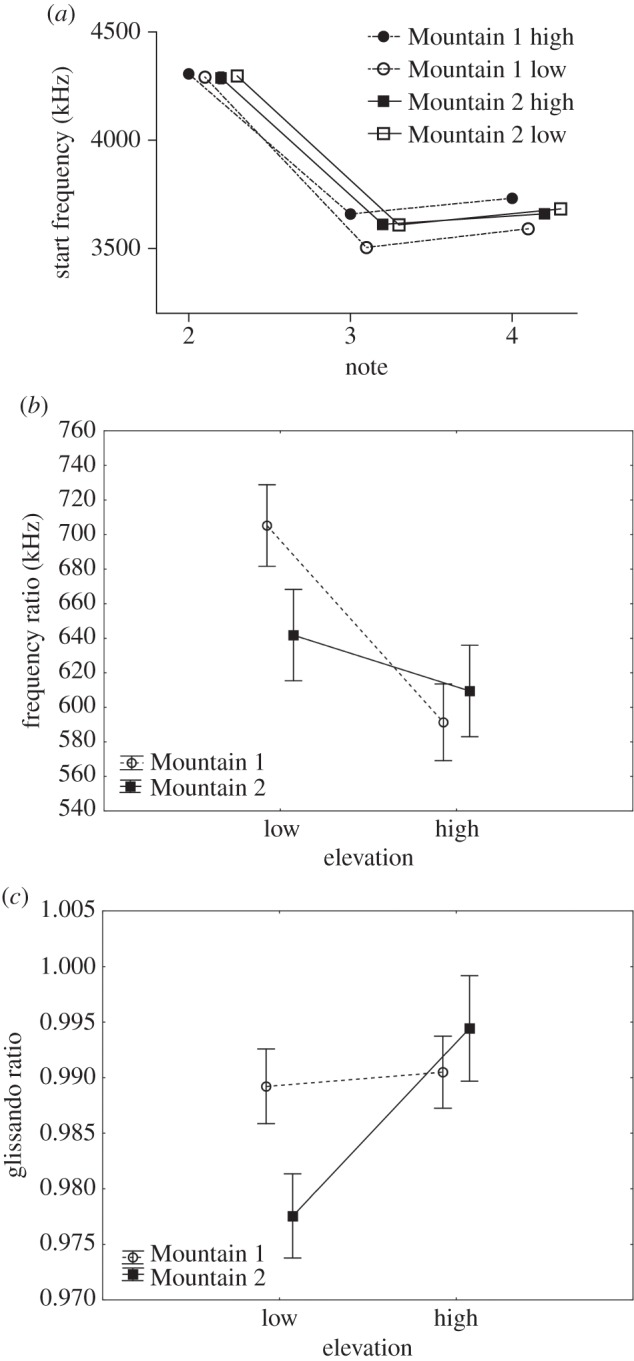
Means and s.e.m. for the three spectral measures that showed significant differences using Tukey post hoc comparisons. (*a*) SF for Notes 2, 3 and 4 from high and low elevation males sampled at Mountains 1 and 2 (note that s.e.m. are present but are very small and therefore do not always appear on the figure), (*b*) FR from high and low elevation males sampled at Mountains 1 and 2, and (*c*) NG for Note 1 from males at high and low elevations sampled from Mountains 1 and 2.

There was no effect for overall location (Mountain 1 versus Mountain 2) (*F*_1,52_=0.3, *p*=0.578) on SF for Notes 2, 3 and 4, however there was a significant difference between elevations (high and low; *F*_1,52_=5.8, *p*=0.02) and a significant interaction between location and elevation (*F*_1,52_=8.3, *p*=0.006; figure 3*a*). The SFs for Notes 3 and 4 between males from high and low elevation were significantly different at Mountain 1, but not at Mountain 2 (table 1). Furthermore, for Note 3 SF, there was a significant difference between Mountain 1 low and Mountain 2 high (*p*=0.043) as well as a trend for difference between Mountain 1 low and Mountain 2 low (*p*=0.054).

There was an effect of overall location (*F*_1,52_=8.3, *p*=0.006), however there was no significant effect of elevation (*F*_1,52_=1.6, *p*=0.212) on the NG of Notes 2, 3 and 4—the interaction between location and elevation was also not significant (*F*_1,52_=0.6, *p*=0.434). There were no significant differences in NG for Notes 2, 3 or 4 between high and low elevations at Mountain 1 or Mountain 2 (table 1), however there was a significant difference in NG for Note 4 between Mountain 1 high and Mountain 2 high (*p*=0.002).

There was no significant effect of overall location (*F*_1,52_=0.84, *p*=0.364), however there was a significant effect of elevation (*F*_1,52_=8.75, *p*=0.005) on FR. The interaction between location and elevation was not significant (*F*_1,52_=2.73, *p*=0.105; figure 3*b*). FRs between males from high and low elevation were significantly different at Mountain 1, but not at Mountain 2 (table 1). There was a significant difference in FR for Mountain 1 low and Mountain 2 high (*p*=0.044).

There was no significant effect of overall location (*F*_1,43_=0.67, *p*=0.419) or elevation (*F*_1,43_=0.72, *p*=0.399) on the SF of Note 1, nor was the interaction between location and elevation significant (*F*_1,43_=0.04, *p*=0.849). There were no significant differences in Note 1 SF for high and low elevation males from either Mountain 1 or Mountain 2 (table 1), and all other comparisons between mountains were not statistically significant.

There was no significant effect of overall location (*F*_1,43_=1.0, *p*=0.319) on the NG of Note 1, however there was a significant effect of elevation (*F*_1,43_=5.6, *p*=0.022) and a significant interaction between location and elevation (*F*_1,43_=4.2, *p*=0.047; figure 3*c*). NG of Note 1 was significantly different between high and low elevation male song from Mountain 2 but not from Mountain 1 (table 1). Differences in NG for Note 1 approached significance between Mountain 1 high and Mountain 2 low (*p*=0.059).

**Figure 4.**
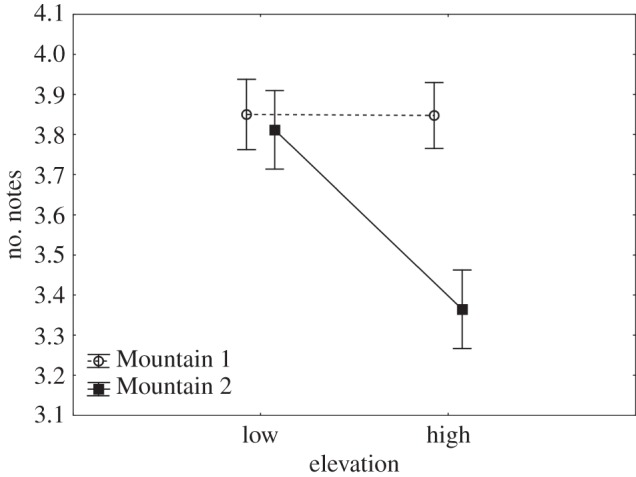
Mean and s.e.m. for NN in songs from high and low elevation males sampled at Mountains 1 and 2.

There was a significant effect of overall location (*F*_1,52_=8.11, *p*=0.006) and elevation (*F*_1,52_=6.02, *p*=0.017) on NN, with a significant interaction between location and elevation (*F*_1,52_=5.89, *p*=0.019; figure 4). There was a significant difference in NN between males at high and low elevations from Mountain 2 but not from Mountain 1 (table 1). There was also a significant difference in NN between Mountain 1 low and Mountain 2 high (*p*=0.003) and Mountain 1 high and Mountain 2 high (*p*=0.002).

#### 4.1.3 Amplitude

**Figure 5.**
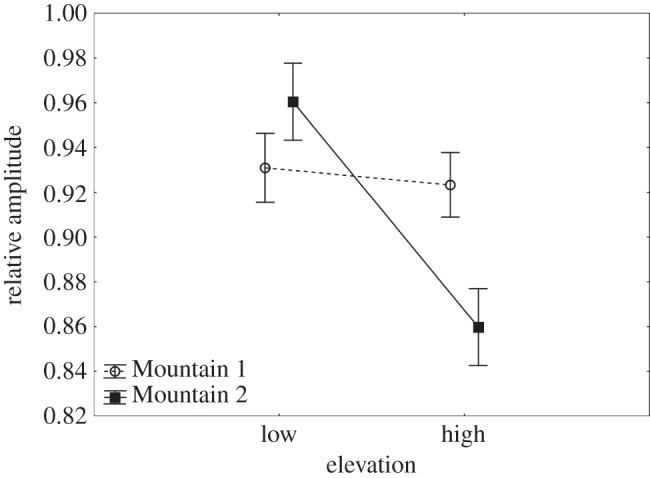
Mean and s.e.m. for RA of male song from high and low elevations sampled at Mountains 1 and 2.

An analysis of the RA, a ratio of the amplitude of Note 3 over Note 2, showed no significant effect of overall location (*F*_1,52_=1.12, *p*=0.295), however there was a significant effect of elevation (*F*_1,52_=11.32, *p*=0.001) and a significant interaction between location and elevation (*F*_1,52_=8.36, *p*=0.006; figure 5). RA for male song from high versus low elevation was significantly different at Mountain 2, but not at Mountain 1 (table 1). However, there was a significant difference between Mountain 1 low and Mountain 2 high (*p*=0.017), as well as a difference between Mountain 1 high and Mountain 2 high (*p*=0.03).

#### 4.1.4 Location-specific individual variation

Only four acoustic measure CVs revealed significant differences between elevations and/or locations. (i) There was an effect of overall location (*F*_1,52_=5.49, *p*=0.023) on NN CV, but no effect of elevation (*F*_1,52_=0.48, *p*=0.49) and no significant interaction between location and elevation (*F*_1,52_=0.13, *p*=0.724). There were no significant differences between high and low elevation in NN CVs at Mountain 1 or Mountain 2, or between mountains (all *p*s>0.05). (ii) There was no significant effect of overall location (*F*_1,52_=0.13, *p*=0.72) on the CV of SF, however there was an effect of elevation (*F*_1,52_=4.11, *p*=0.048), but no significant interaction between location and elevation (*F*_1,52_=1.24, *p*=0.271). There were no significant differences in Notes 2, 3 and 4 SF CVs between high and low elevations from Mountain 1 or Mountain 2, or between mountains (all *p*>0.05). (iii) There was no effect of overall location (*F*_1,43_=0.36, *p*=0.554) on the CV of NG, however there was an effect of elevation (*F*_1,43_=4.68, *p*=0.036), but no significant interaction between location and elevation (*F*_1,43_=1.70, *p*=0.199). Again, there were no significant differences in NG CV for Note 1 between high and low elevation males from Mountain 1 or Mountain 2, or between elevations (all *p*>0.05). (4) Finally, there was no overall effect of location (*F*_1,52_=1.44, *p*=0.236) on the CV of FR, however there was an effect of elevation (*F*_1,52_=11.28, *p*=0.001), with a significant interaction between location and elevation (*F*_1,52_=4.11, *p*=0.048). There was a significant difference in FR CV for males from Mountain 2, such that males from high elevation had a significantly higher FR CV than males from low elevation (*p*=0.004). There were no significant differences in FR CV between high and low elevation males at Mountain 1 (*p*>0.05), however there was a significant difference between Mountain 1 low and Mountain 2 high (*p*=0.013).

### 4.2 Discriminant function analysis

**Figure 6.**
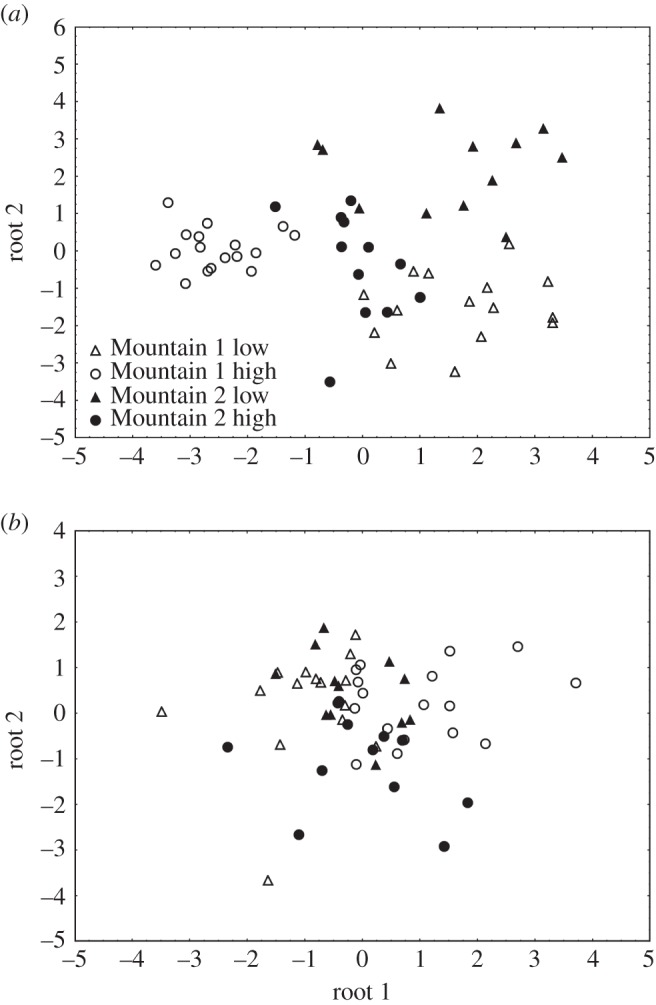
Discriminate function models of 11 variables for high and low elevations sampled at both Mountain 1 and Mountain 2. (*a*) DFA calculated from means, (*b*) DFA calculated from CVs.

We conducted two discriminant function analyses to classify male song based on location (Mountain 1 low and high, Mountain 2 low and high). The first discriminant function analysis classified male songs to a location using the means of 11 acoustic variables (NN, Note 3 SF, FR, Note 2 D, Note 3 D, Note 4 D, Note 3–4 II, Note 2 G, Note 3 G, Note 4 G and RA). This analysis was able to significantly discriminate between songs from the four locations using the 11 acoustic variables (*F*_33,124_=7.94, *p*<0.001). Using the ‘hold-out-sample’ cross-validation method, this DFA correctly assigned 91.67% of the 12 cases (11/12, significant using a binomial test with chance set at 0.50, *p*=0.003; figure 6*a*). The second discriminant function analysis classified male song to a location using the acoustic measure CVs of the same 11 variables listed above; however, this analysis did not discriminate between songs from the four locations (*F*_33,124_=1.47, *p*=0.069). This analysis correctly assigned 66.67% of the 12 cases (8/12, not significant using a binomial test with chance set at 0.50, *p*=0.121) used for cross-validation (figure 6*b*). For the binomial tests, we used a more conservative approach by using a chance level of 0.50 to represent the dichotomy of correct versus incorrect classification; however, we also want to report the binomial test with chance set at 0.25 as there are four possible populations for which a male's song can be classified (both classifications become significant, DFA 1, *p*=0.000 and DFA 2, *p*=0.002). We would, however, like to note that significant classification in DFA 2 at the 0.25 level is driven by one variable, the FR, which differed between Mountain 2 high and low and between Mountain 1 low and Mountain 2 high. See table 2 for eigenvalue and canonical correlation coefficient, as well as the standardized and raw coefficients for the first discriminant function for the discriminant function analyses conducted for means and the CVs.

**Table 2.** The original and cross-validated percentage of correct classifications by group using two ‘all-variables-together’ DFAs, one for means and one for CVs of acoustic parameters measured. The eigenvalue and canonical correlation coefficient, as well as the two acoustic features with the two largest (respectively) standardized and raw coefficients for the first discriminant function are listed for both DFAs, means and CVs.

group	original	cross-validated	eigenvalue	*r*_*c*_	std. coeff	raw coeff
means	94.64	91.67	3.53	0.88	FR, ND of Note 3	NG of Notes 2 and 3
CVs	62.5	66.67	0.59	0.61	NG of Note 2, FR	NG of Notes 2 and 4

## 5. Discussion

**Figure 7.**
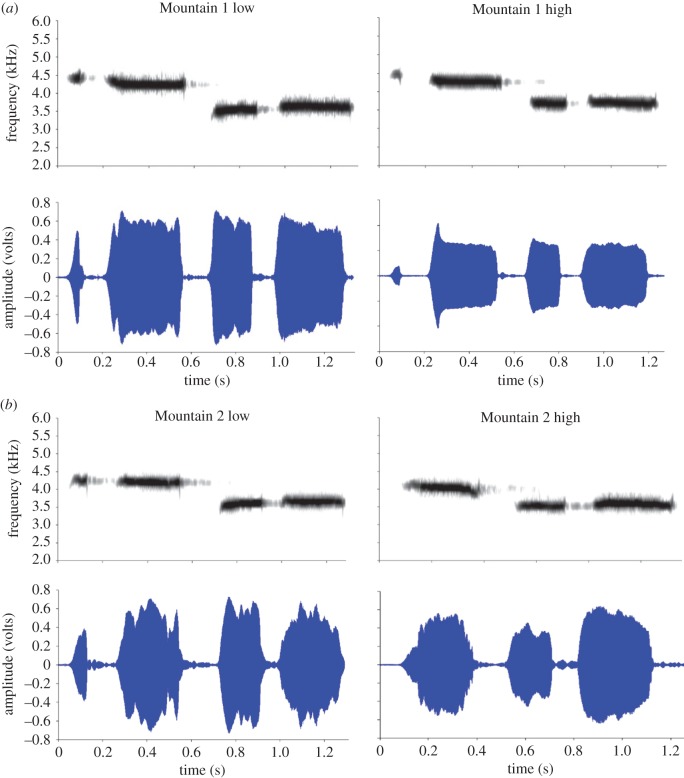
Example of male song from each of the four locations. (*a*) Left, spectrogram and waveform from Mountain 1 low; right, spectrogram and waveform from Mountain 1 high. (*b*) Left, spectrogram and waveform from Mountain 2 low; right, spectrogram and waveform from Mountain 2 high.

Our study showed that mountain chickadees living at high and low elevations from two different mountain locations differ in their song structure. These differences were unique at each of the four locations, and there were no general differences within elevations between the two mountains (see figure 7*a*,*b* for population comparisons). In addition, we found no significant differences in degree of individual variation (CV) among our four sites. Male song did not differ significantly in consistency of production within or between elevations. This finding is inconsistent with the idea that males with superior cognitive abilities are able to produce more consistent songs and therefore may be of higher quality [36–38]. Our high elevation birds have superior cognitive abilities compared with low elevation birds [23,42]; however, unlike previous work suggesting that better quality males sing more consistent songs [36–38], we do not find support for this, at least on a population level.

Several hypotheses could explain differences in male song structure from high and low elevations: (1) the acoustic adaptation hypothesis [10], (2) temporal variation [11] or (3) local adaptation [7,12–14,43] (see Introduction for detailed description). Song in songbirds has been shown to change along an elevation gradient as selection pressures change, including variation in vegetation and/or abundance of heterospecifics, both of which may interfere with signal transmission [44–46]. If song was acoustically adapted (hypothesis 1) to either altitude or elevation-specific habitat, birds at similar elevations should sing similar dialects, while birds at differing elevations should sing different dialects. Both the high and low elevation sites used in this study are found in the Sierra Nevada less than 35 km from each other, and low elevation sites exhibit comparable mixed-conifer forest species composition, and species abundance to each other, as do the two high elevation sites [30]. Despite similarities in within elevation environments, we found no consistent song differences based on differences in elevation across mountain sites; therefore, our results are inconsistent with predictions based on the acoustic adaptation hypothesis. Although we think it is unlikely, it may be possible that some minor specific local differences (besides general forest species and age composition) could contribute to the differences we see between populations.

We did, however, find some support for both hypotheses (2) and (3), as under both hypotheses we predict differences among song collected from each location. However, when combined with previous research, the third hypothesis seems to be the most consistent with our data. Specifically, previous research has shown that mountain chickadees living at high elevations, that experience harsher winter conditions, have superior spatial memory and related brain regions associated with more intense food caching compared with low elevation birds (these results found using different birds from the same Mountain 1 populations at exactly the same elevations used here [23–25]). Given that these birds rely heavily on food caches to survive winter, it seems likely that low elevation birds would be less successful at high elevations due to their lower food caching propensity and inferior memory abilities. Furthermore, high elevation birds are socially subordinate to low elevation birds [26], which may limit high elevation birds from penetrating low elevation, because subordinate birds would experience reduced fitness [47]. The fact that significant differences in song structure are produced on separate mountains along a similar elevation gradient suggests similar selection pressure for locally adapted individuals may lead to such differences. Considering that females might be a driving factor for the evolution of elevation-related song dialects, differences might be expected to evolve independently on different mountains assuming dispersal may occur along the slope of each mountain and not between mountains—chickadees have one dispersal event as juveniles and are thought to disperse rather short distances (0.4–11 km [48]). If this were the case, females at high elevation on each mountain are more likely to encounter a male from low elevation from the same mountain rather than a male from high elevation from a different mountain.

Some research investigating geographical variation in black-capped chickadees focuses on how new dialects arise, via slight alterations or imperfect imitations of song leading to multiple song dialects in newly colonized areas, where individuals are geographically isolated [49,50]. Mountain chickadees in the Sierra Nevada, however, inhabit a continuous gradient of habitat, with no geographical barriers to movement. Despite this continuous distribution, we see significant differences in male song structure. Because song in songbirds is phenotypically plastic, it may be possible for males to move between elevations within a mountain and shift their song to the local structure (vocal fluctuation in response to noise [51–53]); however, this is unlikely because: (i) song in chickadees is learned from the natal habitat from other local males; once males disperse they may prune their repertoire, however once it is pruned it becomes crystallized [54]. (ii) If birds are able to move to another elevation or location and shift their songs to the local dialect it would not serve as an honest cue [55] of location and therefore females, as a general rule, should have no preference for local dialects [7,8]. Finally (iii) if males were moving between elevations or across mountains and shifting their song, we would not have detected the differences in song structure between high and low elevation male song across the two mountains sampled here.

If high and low elevation birds are locally adapted to their respective elevations (differences in spatial memory, food caching and exploration rates), it would benefit females to be able to discriminate between males from different elevations in order to mate with those from their respective elevation to produce the most fit offspring. Our recent work has shown that in a pairwise choice of high and low elevation males, high elevation females prefer high elevation males to low elevation males [29], despite their socially subordinate status (a feature known to be important in mate acquisition and fitness [47]). It is unknown what cue or proxy females may use to discriminate between males from high and low elevations; however, the plethora of differences seen here in song structure suggest that song may provide a salient signal of location of origin, which could be used in discrimination in the wild. Future research will address females' ability to discriminate between male song from high and low elevations and their potential preference for these different song types.

In order to show support for hypothesis (2), we would need to show that there is genetic population structure between the four locations sampled here. Preliminary data from our laboratory has sampled these exact same populations and suggests no genetic population structure. However, even without this preliminary data, it would seem unlikely that there is absolutely no gene flow or movement between these four locations because there is a continuous distribution of chickadees along each mountain, with only a few kilometres between the elevations sampled. In addition, the two mountains sampled are less than 35 km away from one another, and there is no geographical barrier preventing movement, granted chickadees are known to disperse rather short distances [48]. Movements between elevations are probably somewhat restricted due to local adaptations; however, variation in climate severity among years may allow some movement between elevations, especially in years with mild winters. Even a low amount of movement between elevations might be sufficient to prevent genetic population structure [56], moreover local adaptations are known to evolve even in the face of gene flow [20,57].

Future work will address song structure across a gradient of elevations using these same mountains in order to elucidate if the differences in song observed here are a result of clinal variation or if they represent true ‘high’ and ‘low’ elevation dialects. An abrupt change in song structure would be suggestive of song dialects and would further suggest limited gene flow between elevations. If there is an elevation location where one dialects ends and the other begins, that location will inform future research related to high and low elevation contact zones and change in spatial memory ability needed to retrieve food caches. This information may help us answer whether these groups of individuals are shifting into separate populations or if there is continuous mixing along the high and low elevation gradient. This question is particularly interesting given the data correlating song dialects and gene flow. While some research suggests that song dialects represent a reduction in gene flow [14,58], an abundance of work has found differences in song structure despite gene flow [59–61]. Given the local adaptations we see in mountain chickadees and differences in song structure found here, it is critical to understand the levels of gene flow between high and low elevation as well as between mountains to identify the specific mechanism generating this variation in song structure.

## Supplementary Material

Acoustic Measurements for Branch & Pravosudov

## Data accessibility

All data are available in the electronic supplementary information.
